# Proton Pump Inhibitor Use and Clinical Outcomes in Atrial Fibrillation During Anticoagulation

**DOI:** 10.3390/jcm15103590

**Published:** 2026-05-08

**Authors:** Do-Young Kim, Hye Young Lee, Eileen Laurel Yoon, Seung-Young Roh, Kwang-No Lee

**Affiliations:** 1 Department of Cardiology, Ajou University School of Medicine, World Cup-ro 164, Yeongtong-gu, Suwon 16499, Republic of Korea; 2 Division of Cardiology, Department of Internal Medicine, Sanggye-Paik Hospital, Inje University College of Medicine, Dongil-ro 1342, Nowon-gu, Seoul 01757, Republic of Korea; 3 Division of Gastroenterology and Hepatology, Department of Internal Medicine, Hanyang University Hospital, Wangsimni-ro 222-1, Seongdong-gu, Seoul 04763, Republic of Korea; 4 Division of Cardiology, Department of Internal Medicine, Korea University Guro Hospital, Korea University College of Medicine, Gurodong-ro 148, Guro-gu, Seoul 08308, Republic of Korea

**Keywords:** atrial fibrillation, oral anticoagulants, proton pump inhibitors, gastrointestinal bleeding, time-varying exposure

## Abstract

**Purpose**: Proton pump inhibitors are commonly used during oral anticoagulant therapy in patients with atrial fibrillation, but evidence regarding outcomes beyond upper gastrointestinal bleeding remains limited. We evaluated whether concomitant proton pump inhibitor use during oral anticoagulant therapy was associated with thromboembolic events, bleeding outcomes, and all-cause mortality. **Methods**: This retrospective multicenter cohort study included patients with atrial fibrillation who initiated oral anticoagulant therapy. Concomitant proton pump inhibitor use was modeled as a time-varying exposure with a prespecified 7-day lag. The primary outcome was a composite of thromboembolic events, major bleeding, and all-cause mortality. Secondary outcomes included each component outcome and gastrointestinal bleeding. Associations were estimated using time-dependent Cox proportional hazard models after multiple imputation of missing baseline variables. **Results**: Among 11,203 patients (median age 71 years [interquartile range 62–78]; 4743 women [42.3%]), 7-day lagged time-varying proton pump inhibitor exposure was associated with a higher risk of the composite outcome (hazard ratio 1.29, 95% confidence interval 1.08–1.55), major bleeding (1.80, 1.36–2.37), gastrointestinal bleeding (1.77, 1.18–2.66), and all-cause mortality (1.58, 1.00–2.48). No statistically significant association was observed for thromboembolic events. Across robustness analyses, the overall pattern was broadly maintained, although estimates varied according to exposure timing. **Conclusions**: In this observational cohort of patients with atrial fibrillation receiving oral anticoagulant therapy, concomitant proton pump inhibitor use modeled with a 7-day lagged time-varying framework was associated with higher risks of several bleeding-related outcomes and all-cause mortality, but not thromboembolism. These findings should be interpreted as associations rather than causal effects.

## 1. Introduction

Atrial fibrillation is the most common sustained cardiac arrhythmia and is associated with a substantially increased risk of stroke, systemic thromboembolism, heart failure, and death. The current major guidelines recommend oral anticoagulant therapy for eligible patients with atrial fibrillation to reduce thromboembolic risk [1,2].

Bleeding remains a major limitation of oral anticoagulant therapy, particularly in older patients with multiple comorbidities, and the gastrointestinal tract is among the most clinically important bleeding sites during treatment [1,2,3]. Proton pump inhibitors are therefore often used as gastroprotective therapy in patients considered to be at increased risk of upper gastrointestinal bleeding. Recent observational studies and population-based analyses have continued to focus mainly on upper gastrointestinal bleeding and have generally suggested that proton pump inhibitor cotherapy may be associated with a lower risk of that outcome during oral anticoagulant treatment [3,4,5]. However, evidence remains limited regarding broader clinical outcomes, including thromboembolic events, major bleeding beyond the upper gastrointestinal tract, site-specific gastrointestinal bleeding outcomes, and all-cause mortality [5,6].

In addition, interpretation of concomitant proton pump inhibitor use during ongoing oral anticoagulant therapy is methodologically challenging [7]. In clinical practice, proton pump inhibitors may be initiated in response to early gastrointestinal symptoms, perceived bleeding risk, or clinical deterioration shortly before an outcome event; so, baseline-fixed or temporally naïve exposure definitions may be vulnerable to time-related bias, exposure misclassification, and reverse causation [6,8]. Accordingly, we conducted a retrospective multicenter cohort study to evaluate whether concomitant proton pump inhibitor use during oral anticoagulant therapy in patients with atrial fibrillation was associated with thromboembolic events, bleeding outcomes including gastrointestinal bleeding, and all-cause mortality, using a long-format time-varying analytical framework.

## 2. Materials and Methods

### 2.1. Data Source

This retrospective multicenter cohort study used electronic medical record data from four tertiary referral hospitals in South Korea. The database included diagnostic codes, prescription records, procedure and billing data, and laboratory test results. This study was conducted in accordance with the Declaration of Helsinki and was approved by the institutional review boards of all participating hospitals. The requirement for informed consent was waived because of the retrospective design. All patient data were anonymized before analysis.

### 2.2. Study Design and Cohort

**Figure 1:**
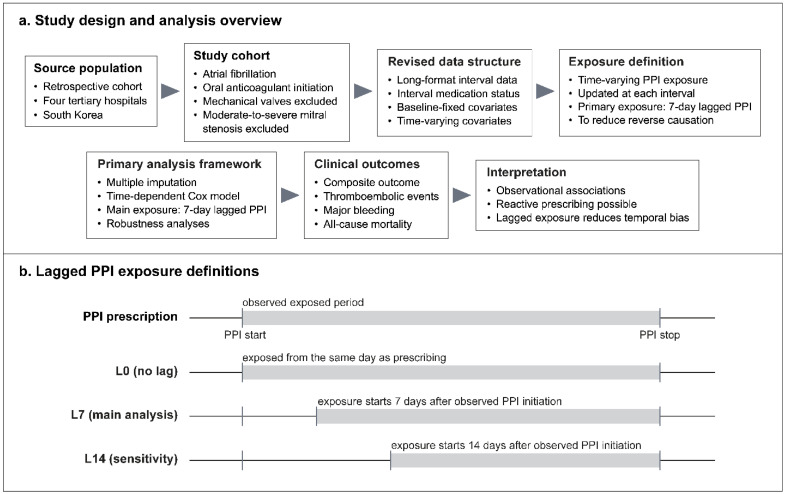
Study flowchart and exposure lag schema. Study flowchart and primary analytic framework (**a**). Comparison of L0, L7, and L14 definitions for time-varying proton pump inhibitor exposure (**b**). The primary analysis used L7.

The study cohort comprised patients with atrial fibrillation who initiated oral anticoagulant therapy with warfarin, dabigatran, rivaroxaban, apixaban, or edoxaban between 1 January 2013 and 31 March 2020 (Figure 1a). Patients with mechanical heart valves and those with documented moderate-to-severe mitral stenosis were excluded. This study used an episode-based long-format data structure in which each oral anticoagulant treatment episode was divided into sequential start-stop intervals during follow-up. Within this structure, proton pump inhibitor exposure and other time-varying medication variables were updated at each interval, whereas baseline characteristics were retained as fixed covariates.

For each eligible treatment episode, follow-up began on the episode start date and continued until the occurrence of the outcome of interest, discontinuation of oral anticoagulant therapy defined as a gap of more than 30 days without prescription refill, or the end of study follow-up. Oral anticoagulant exposure windows were constructed from prescription dates and days supplied. To account for residual pharmacologic effect, the exposure period was extended beyond the supplied days by 1 day for non-vitamin K antagonist oral anticoagulants and by 3 days for warfarin.

### 2.3. Exposure Assessment

The main exposure of interest was concomitant proton pump inhibitor use during oral anticoagulant therapy. Proton pump inhibitor exposure was treated as a time-varying variable that was updated across follow-up intervals rather than as a single exposure status defined at cohort entry (Figure 1b). The primary exposure definition applied a 7-day lag to proton pump inhibitor use during follow-up. This lagged exposure definition was chosen to reduce bias arising from proton pump inhibitor initiation shortly before clinical events. The 7-day interval was not intended to represent a pharmacological latency period, but was prespecified as a pragmatic temporal buffer reflecting real-world prescribing patterns, in which early symptoms, perceived bleeding risk, or clinical deterioration may prompt proton pump inhibitor prescribing immediately before an outcome event.

Additional exposure definitions were examined in sensitivity analyses. These included a no-lag definition and a 14-day lag definition to assess whether the estimated associations varied according to the temporal definition of proton pump inhibitor exposure. A separate sensitivity analysis examined a simplified specification in which proton pump inhibitor use and concomitant medication use were defined only at cohort entry and then kept unchanged throughout follow-up, rather than being updated over time.

In the time-dependent Cox models, concomitant proton pump inhibitor exposure was defined by interval-specific overlap status during follow-up rather than by the cumulative duration of overlap across the treatment episode.

### 2.4. Outcomes

The primary outcome was a composite of thromboembolic events, major bleeding, and all-cause mortality. Secondary outcomes included each individual component of the composite outcome and gastrointestinal bleeding.

Thromboembolic events included ischemic stroke, systemic embolism involving the renal, mesenteric, ophthalmic, or extremity arteries, and left-sided intracardiac thrombus confirmed by radiographic or sonographic imaging. Ischemic stroke required supporting neuroimaging findings. Events initially diagnosed at outside hospitals were included only when documented by official diagnostic reports.

Major bleeding was defined according to the International Society on Thrombosis and Hemostasis criteria for major bleeding in non-surgical patients [9]. Candidate bleeding events were identified from electronic medical record data, including diagnostic codes, procedure or billing records, laboratory data, transfusion records, endoscopic or imaging reports, and clinical documentation when available. Bleeding events were not classified solely on the basis of diagnostic codes, and available source documentation was reviewed at each participating center according to the prespecified study definitions. Previous validation work in patients receiving oral anticoagulation has shown that International Classification of Diseases, 10th Revision code-based algorithms for gastrointestinal bleeding can have high positive predictive value, but that sensitivity may be improved by reviewing endoscopic results and other source documentation [10]. Gastrointestinal bleeding was evaluated separately, including total gastrointestinal bleeding and site-specific subtypes. Subtypes were classified according to the documented bleeding source in available clinical, endoscopic, imaging, procedure, and transfusion-related records. Upper gastrointestinal bleeding was defined as bleeding from the esophagus, stomach, or duodenum, and lower gastrointestinal bleeding was defined as bleeding from a colorectal source [11]. Events in which the bleeding source could not be clearly localized from the available records were classified as gastrointestinal bleeding of undetermined source.

### 2.5. Covariates and Missing Data

Covariates included baseline characteristics, selected laboratory variables, and concomitant medications. Baseline characteristics and selected laboratory variables were defined at cohort entry and retained unchanged throughout follow-up. Concomitant medication variables were updated across follow-up intervals when applicable.

Because some baseline variables had missing values, multiple imputation was performed before model fitting. The imputation model was specified separately from the outcome model. Variables entered into the imputation model were selected to preserve the relationships among exposure, covariates, and outcomes, whereas the outcome model included prespecified covariates used for confounding control in the time-dependent survival analyses. Estimates from the imputed datasets were combined using Rubin’s rules [12]. Variable level missingness and summaries of observed and imputed values in the primary analysis cohort and in the restricted cohort without prior proton pump inhibitor exposure are provided in Table S1 and Table S2, respectively.

### 2.6. Statistical Analysis

Continuous variables were summarized as mean with standard deviation or median with interquartile range, as appropriate, and categorical variables as number with percentage.

The primary analysis used a time-dependent Cox proportional hazard model in the long-format dataset after multiple imputation of baseline variables with missing values. Hazard ratios and 95% confidence intervals were estimated for the primary and secondary outcomes. For non-fatal outcomes, death was treated as a competing event through cause-specific censoring. The proportional hazard assumption was assessed using scaled Schoenfeld residuals for the primary time-dependent Cox models across the imputed datasets.

Additional analyses were performed to examine the robustness of the findings under alternative analytic assumptions. Stabilized inverse probability of treatment weighting at the interval level was used as an alternative approach to confounding control and to assess robustness to measured time-varying confounding related to interval-updated concomitant medication use. Covariate balance after weighting was evaluated using absolute standardized mean differences summarized across imputed datasets. No-lag and longer-lag exposure definitions were evaluated to assess the sensitivity of the estimates to the temporal specification of proton pump inhibitor exposure. A 30-day landmark analysis, in which follow-up was restricted to patients remaining at risk 30 days after cohort entry, was performed to assess whether early follow-up disproportionately influenced the main results. An analysis in patients without proton pump inhibitor prescriptions during the 6 months before oral anticoagulant initiation was conducted to examine whether the findings were sensitive to recent proton pump inhibitor use before cohort entry. A complete case analysis without multiple imputation was performed to assess the influence of missing-data handling. An additional analysis defined proton pump inhibitor use and concomitant medication use only at cohort entry and did not update them during follow-up, to evaluate the impact of simplifying time-varying medication exposure. Because treatment with proton pump inhibitors was not randomly assigned, these analyses were also used to assess the robustness of the findings to measured confounding, alternative exposure definitions, early follow-up, prior proton pump inhibitor exposure, and missing-data handling.

Exploratory subgroup analyses and tests for interaction were also performed. In addition, a separate descriptive analysis examined whether first proton pump inhibitor initiation clustered shortly before outcome events to support the choice of the 7-day lag. This analysis was intended to inform the exposure definition and not to estimate treatment effects.

All analyses were performed using R version 4.5.2 in RStudio version 2026.01.1 Build 403. Reporting of this cohort study followed the Strengthening the Reporting of Observational Studies in Epidemiology guidelines, and the completed checklist is provided in Table S4.

## 3. Results

### 3.1. Study Population

A total of 11,203 patients with atrial fibrillation receiving oral anticoagulant therapy were included. Baseline proton pump inhibitor users comprised 28.7% of the cohort and were generally older and clinically more complex than non-users (Table 1). Because the main exposure was defined as a lagged time-varying proton pump inhibitor variable, these baseline comparisons are descriptive and do not represent the analytic contrast used for effect estimation. Missing data were primarily present in several anthropometric and laboratory variables, and the observed and imputed distributions were broadly comparable, supporting the use of multiple imputation (Table S1 and Figure S1).

### 3.2. Primary Analysis

In the primary analysis using 7-day lagged time-varying proton pump inhibitor exposure, concomitant proton pump inhibitor use was associated with higher risks of the composite outcome, several bleeding-related outcomes, and all-cause mortality (Table 2). No statistically significant association was observed for thromboembolism or upper gastrointestinal bleeding.

### 3.3. Robustness Analyses

**Figure 2:**
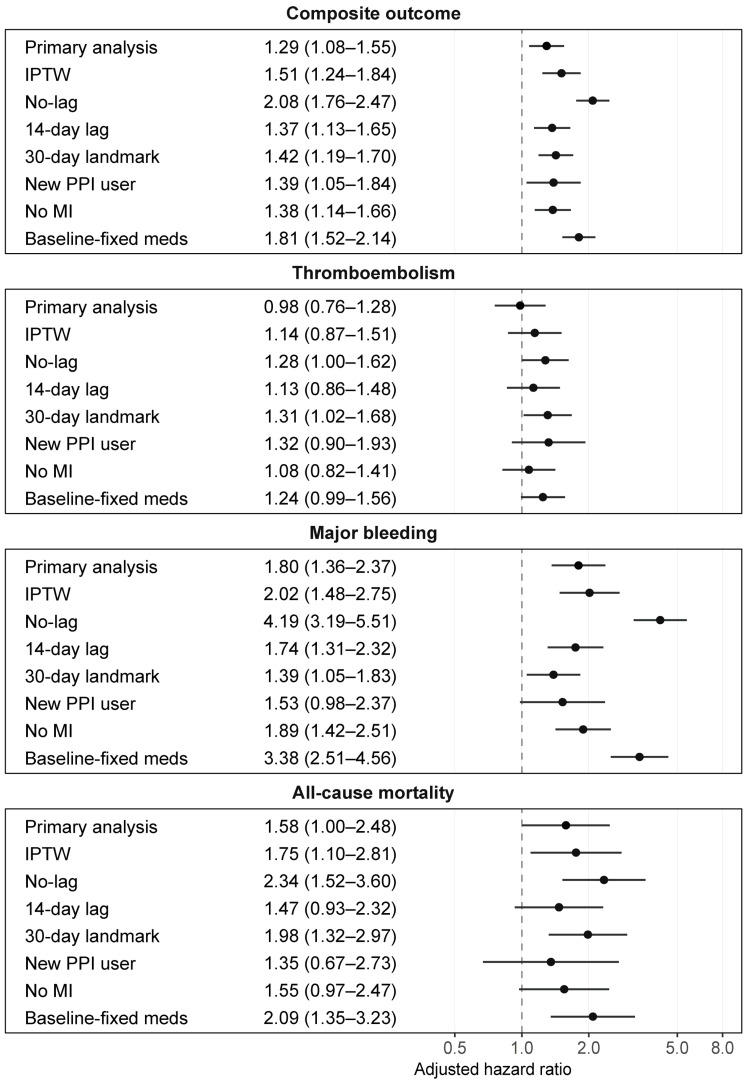
Robustness of association estimates across clinical outcomes. Adjusted hazard ratios and 95% confidence intervals for the association between concomitant proton pump inhibitor use and four clinical outcomes during oral anticoagulant therapy. The primary analysis is shown together with robustness analyses using alternative analytic strategies. Overall consistency in the direction and magnitude of estimates supports robustness across analyses. Analysis labels: IPTW, inverse probability of treatment weighting; No-lag, unlagged exposure definition; 14-day lag, 14-day lagged exposure definition; 30-day landmark, analysis excluding the first 30 days; New PPI user, analysis restricted to patients with new proton pump inhibitor initiation; No MI, analysis without multiple imputation; Baseline-fixed meds, analysis using baseline-fixed medication covariates.

Across the robustness analyses, the overall pattern of positive associations for the composite outcome and several bleeding-related outcomes was largely maintained (Figure 2 and Figure S2). In the weighting-based analysis, the love plot showed improved balance in measured covariates after stabilized inverse probability of treatment weighting, and the weighted analysis showed directions of association similar to those of the primary analysis (Figure S3). In the lag sensitivity analyses, estimates were larger with the no-lag definition and were attenuated with the 14-day lag definition, particularly for bleeding outcomes.

In the 30-day landmark analysis, positive associations remained for the composite outcome, major bleeding, all-cause mortality, lower gastrointestinal bleeding, and gastrointestinal bleeding of undetermined source, whereas total and upper gastrointestinal bleeding were not statistically significant. In the restricted cohort without prior proton pump inhibitor exposure and in the complete-case analyses without multiple imputation, the overall pattern was broadly similar to that of the primary analysis, although precision was lower for individual outcomes and the association with all-cause mortality was attenuated in the complete-case analysis.

In contrast, the baseline-fixed medication analysis yielded generally larger estimates across multiple outcomes than the primary analysis.

### 3.4. Model Diagnostics

The proportional hazard assumption was assessed using scaled Schoenfeld residuals for the primary time-dependent Cox models (Table S3). Possible non-proportionality for the main proton pump inhibitor exposure was observed for the composite outcome and thromboembolic outcome models, whereas no consistent violation signal was observed for major bleeding, gastrointestinal bleeding outcomes, or all-cause mortality. Therefore, the hazard ratios for the composite and thromboembolic outcomes should be interpreted as overall summary associations across follow-up rather than as strictly time-constant associations.

### 3.5. Subgroup Analyses

In exploratory subgroup analyses, the direction of association for the composite outcome, major bleeding, and all-cause mortality was generally consistent across most subgroups, whereas findings for thromboembolism were more heterogeneous (Figure S4). Formal evidence of interaction was limited to selected subgroups, and there was no strong overall evidence of marked heterogeneity.

### 3.6. Assessment of the 7-Day Lag Definition

**Figure 3:**
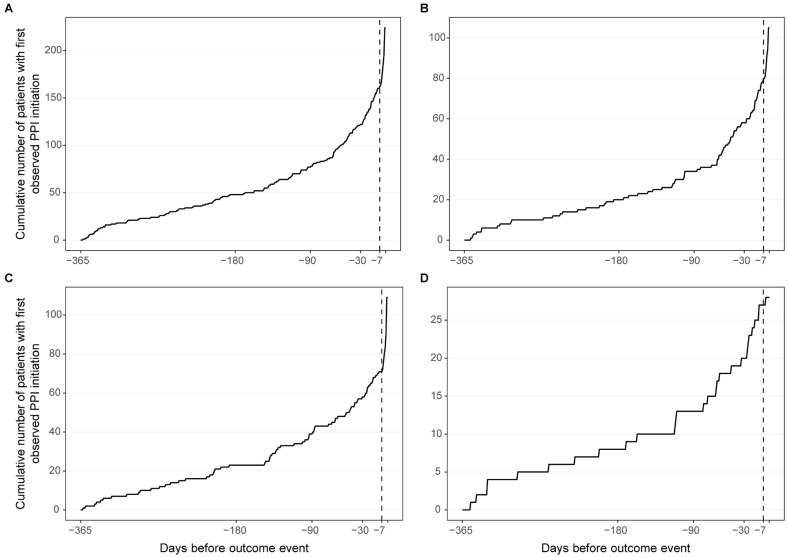
Event-time clustering of first observed proton pump inhibitor initiation before clinical outcomes. Cumulative number of patients with a first observed proton pump inhibitor initiation within 365 days before the outcome event for the composite outcome (**A**), thromboembolism (**B**), major bleeding (**C**), and all-cause mortality (**D**). The dashed vertical line marks day −7, corresponding to the primary lag used in the primary analysis. These plots are presented to support the lagged exposure definition, not to estimate treatment effects.

Event-time plots of first observed proton pump inhibitor initiation before outcome occurrence are shown in Figure 3 and Figure S5. These plots were included to assess whether proton pump inhibitor initiation clustered shortly before outcome events, thereby supporting the use of a lagged exposure definition in the primary analysis. Any clustering of first proton pump inhibitor initiation shortly before outcome occurrence should be interpreted as a marker of exposure timing and potential time-related bias, rather than as evidence of a treatment effect.

## 4. Discussion

In this multicenter retrospective cohort of patients with atrial fibrillation receiving oral anticoagulant therapy, concomitant proton pump inhibitor use defined with a 7-day lagged time-varying framework was associated with higher overall hazards of the composite outcome, several bleeding-related outcomes, and all-cause mortality, whereas no clear overall association was observed for thromboembolism. The direction of association was broadly maintained across robustness analyses, although estimates varied according to exposure timing and analytic specification. These findings suggest that temporal handling of proton pump inhibitor exposure was important and that the results should be interpreted as observational associations within a dynamic prescribing context.

This sensitivity to exposure timing is clinically and methodologically important. In routine practice, proton pump inhibitors may be initiated in response to early gastrointestinal symptoms, perceived bleeding risk, or clinical deterioration shortly before an outcome event. Accordingly, proton pump inhibitor exposure recorded shortly before the outcome may partly reflect reactive prescribing and underlying patient risk rather than a direct detrimental effect of the drug itself. The 7-day lag should therefore be interpreted as a design choice to reduce short-term reverse causation and protopathic bias, rather than as a pharmacological assumption about the timing of proton pump inhibitor effects. Recent methodological work has emphasized that misalignment of eligibility, exposure assignment, and start of follow-up can introduce important bias in observational treatment studies [13,14]. In addition, confounding by indication and protopathic bias may distort apparent associations when treatment is initiated in higher-risk patients or in response to early manifestations of the outcome process [15,16,17]. Viewed in this context, the larger estimates in the no-lag analysis and the attenuation seen with the 14-day lag definition suggest that the estimated associations were sensitive to the temporal definition of exposure. This pattern supports the use of a lagged exposure definition in the primary analysis, while also indicating that the magnitude of the observed associations should be interpreted cautiously in relation to prescribing context and patient risk characteristics.

These findings should be interpreted in the context of prior studies, which have mainly focused on upper gastrointestinal bleeding and have generally suggested a lower risk of that outcome among oral anticoagulant users receiving proton pump inhibitors [3,4,18]. In the present study, upper gastrointestinal bleeding was the only gastrointestinal bleeding subtype with a directionally lower, although imprecise and statistically nonsignificant, association. This pattern is clinically plausible because acid suppression would be expected to have its most direct gastroprotective effect in the upper gastrointestinal tract [3,19,20].

The clinical implication is not that proton pump inhibitors should be avoided in patients receiving oral anticoagulants. Rather, concomitant proton pump inhibitor use should be interpreted as a marker of a higher-risk prescribing context, particularly when observed near outcome events. Clinicians should continue to use gastroprotective therapy when clinically indicated, while recognizing that proton pump inhibitor initiation during anticoagulant therapy may identify patients with gastrointestinal symptoms, prior bleeding risk, multimorbidity, frailty, or clinical deterioration. Therefore, the observed associations should be used to inform risk interpretation and future study design, not to infer a direct harmful effect of proton pump inhibitors.

Several limitations should be acknowledged. First, residual confounding remains possible in this retrospective observational study. Proton pump inhibitor use may reflect prior bleeding, gastrointestinal symptoms, concomitant antiplatelet or nonsteroidal anti-inflammatory drug use, multimorbidity, frailty, or clinician-perceived bleeding risk. Therefore, confounding by indication, symptom-driven prescribing, and other time-related biases may have persisted despite the lagged time-varying design, multivariable adjustment, interval-level weighting, and complementary sensitivity analyses [13,14,15,16]. Second, proton pump inhibitor exposure was ascertained from prescription records and may not fully capture actual intake, adherence, or nonprescription use [16]. This limitation may be particularly relevant in health care systems where nonprescription access to acid-suppressive therapy is common. Third, possible non-proportionality was observed for the composite and thromboembolic outcome models, indicating that the corresponding hazard ratios should be interpreted as overall summary associations across follow-up rather than as strictly time-constant associations. Fourth, some outcome-specific and subgroup-specific analyses were limited by reduced precision. Finally, although the lagged time-varying approach was intended to better align exposure timing with follow-up and reduce short-term reverse causation, it cannot eliminate all bias inherent to nonrandomized treatment comparisons [13,14,16]. These findings may be most applicable to clinical settings with similar patient characteristics, anticoagulation practice, health care access, prescription-based medication capture, and proton pump inhibitor prescribing patterns. Generalizability to other populations should be considered cautiously because gastroprotective strategies during oral anticoagulant therapy, reimbursement systems, and access to nonprescription acid-suppressive therapy may differ across countries and health care systems.

## 5. Conclusions

Among patients with atrial fibrillation receiving oral anticoagulant therapy, concomitant proton pump inhibitor use modeled with a 7-day lagged time-varying framework was associated with higher risks of several bleeding-related outcomes and all-cause mortality, whereas no clear association was observed for thromboembolism. These observational findings should not be interpreted as causal evidence of harm from proton pump inhibitors. The sensitivity of these estimates to exposure timing suggests that prescribing context, underlying patient risk, and time-related bias are central to interpretation.

## Figures and Tables

**Table 1:** Baseline characteristics of the study cohort.

Variables	Overall	Proton Pump Inhibitor at Baseline		Standardized Mean Difference
		Yes	No	
Number of patients	11,203	3211	7992	
Age, years	71.0 [62.0, 78.0]	74.0 [65.0, 80.0]	70.0 [61.0, 77.0]	0.282
Female	4743 (42.3)	1492 (46.5)	3251 (40.7)	0.117
Institution				0.241
1	3784 (33.8)	911 (28.4)	2873 (35.9)	
2	2456 (21.9)	807 (25.1)	1649 (20.6)	
3	3536 (31.6)	942 (29.3)	2594 (32.5)	
4	1427 (12.7)	551 (17.2)	876 (11.0)	
Year of first oral anticoagulant use				0.338
2013	869 (7.8)	133 (4.2)	736 (9.2)	
2014	1373 (12.3)	250 (7.8)	1123 (14.1)	
2015	1374 (12.3)	376 (11.7)	998 (12.5)	
2016	1466 (13.1)	455 (14.2)	1011 (12.7)	
2017	1870 (16.7)	555 (17.3)	1315 (16.5)	
2018	1823 (16.3)	548 (17.1)	1275 (16.0)	
2019	1988 (17.7)	720 (22.4)	1268 (15.9)	
2020	440 (3.9)	174 (5.4)	266 (3.3)	
Height, cm	163.0 [155.0, 170.0]	162.0 [155.0, 169.4]	163.8 [155.1, 170.0]	0.132
Weight, kg	64.0 [55.8, 72.3]	62.0 [54.0, 70.5]	65.0 [56.6, 73.0]	0.198
CHA_2_DS_2_-VASc score	2 [1, 3]	2 [1, 3]	2 [1, 3]	0.479
Heart failure	2975 (26.6)	969 (30.2)	2006 (25.1)	0.114
Hypertension	5226 (46.6)	1343 (41.8)	3883 (48.6)	0.136
Diabetes mellitus	2205 (19.7)	649 (20.2)	1556 (19.5)	0.019
Dyslipidemia	1485 (13.3)	381 (11.9)	1104 (13.8)	0.058
Peripheral arterial disease	123 (1.1)	54 (1.7)	69 (0.9)	0.073
Coronary artery disease or myocardial infarction	976 (8.7)	357 (11.1)	619 (7.7)	0.116
Liver cirrhosis	156 (1.4)	59 (1.8)	97 (1.2)	0.051
Prior thromboembolic event ^1^	2686 (24.0)	1235 (38.5)	1451 (18.2)	0.463
Prior bleeding event ^2^	480 (4.3)	199 (6.2)	281 (3.5)	0.125
Hematocrit, %	39.8 [35.0, 43.9]	37.7 [32.7, 42.1]	40.7 [36.3, 44.5]	0.434
Platelet count, ×10^9^/L	198.0 [160.0, 242.0]	195.0 [154.0, 240.0]	200.0 [162.2, 242.0]	0.053
Aspartate aminotransferase, IU	26.0 [21.0, 34.0]	27.0 [21.0, 37.0]	26.0 [21.0, 33.0]	0.102
Alanine aminotransferase, IU	20.0 [14.0, 30.0]	19.0 [13.0, 30.0]	20.0 [14.0, 30.0]	0.075
Fibrosis-4 index for liver fibrosis	2.12 [1.48, 3.11]	2.33 [1.60, 3.53]	2.03 [1.43, 2.92]	0.134
Total bilirubin, mg/dL	0.7 [0.5, 1.0]	0.7 [0.5, 1.0]	0.7 [0.5, 1.0]	0.024
Albumin, g/dL	4.0 [3.7, 4.3]	3.9 [3.4, 4.2]	4.1 [3.8, 4.4]	0.438
Creatinine, mg/dL	0.87 [0.72, 1.06]	0.85 [0.69, 1.06]	0.88 [0.73, 1.06]	0.032
Estimated glomerular filtration rate ^3^, mL/min/1.73 m^2^	85.6 [67.6, 95.7]	85.3 [65.5, 95.2]	85.6 [68.3, 95.8]	0.076
Non-vitamin K oral anticoagulant at baseline	6835 (61.0)	2148 (66.9)	4687 (58.6)	0.171
Antiplatelet drug at baseline	3540 (31.6)	1365 (42.5)	2175 (27.2)	0.325
Diuretic drug at baseline	4069 (36.3)	1500 (46.7)	2569 (32.1)	0.302
Nonsteroidal anti-inflammatory drug at baseline	1557 (13.9)	831 (25.9)	726 (9.1)	0.453
Renin-angiotensin system inhibitor at baseline	4284 (38.2)	1221 (38.0)	3063 (38.3)	0.006
Beta-blocker at baseline	4390 (39.2)	1366 (42.5)	3024 (37.8)	0.096
Statin at baseline	4364 (39.0)	1650 (51.4)	2714 (34.0)	0.358

Categorical variables are presented as number (percentage). Continuous variables listed by the script as nonnormal are presented as median [interquartile range]. ^1^ Prior thromboembolic event included ischemic stroke, transient ischemic attack, myocardial infarction, intracardiac thrombus, systemic embolism, deep vein thrombosis, and pulmonary embolism. ^2^ Prior bleeding event included bleeding events corresponding to the International Society on Thrombosis and Haemostasis criteria for major bleeding. ^3^ Estimated glomerular filtration rate was calculated using the Chronic Kidney Disease Epidemiology Collaboration 2021 equation.

**Table 2:** Primary analysis of 7-day lagged time-varying proton pump inhibitor exposure during oral anticoagulant therapy and clinical outcomes.

Outcome	Events	Person-Years	Incidence Rate (/100 Person-Year)	Unadjusted Hazard Ratio (95% Confidence Interval)	*p* Value	Adjusted Hazard Ratio (95% Confidence Interval) ^1^	*p* Value
Composite outcome	705	13,256	5.32	1.90 (1.60–2.26)	<0.001	1.29 (1.08–1.55)	0.005
Thromboembolic event	400	13,412	2.98	1.43 (1.11–1.83)	0.005	0.98 (0.76–1.28)	0.900
Major bleeding event	245	13,632	1.80	2.69 (2.05–3.52)	<0.001	1.80 (1.36–2.37)	<0.001
Gastrointestinal, total	127	13,817	0.92	2.68 (1.83–3.91)	<0.001	1.77 (1.18–2.66)	0.006
Upper	41	13,731	0.30	1.24 (0.56–2.75)	0.597	0.74 (0.33–1.65)	0.465
Lower	34	13,790	0.25	4.25 (2.14–8.44)	<0.001	2.64 (1.30–5.38)	0.007
Undetermined	85	13,800	0.62	3.39 (2.18–5.27)	<0.001	2.48 (1.51–4.08)	<0.001
All-cause mortality	109	13,750	0.79	2.31 (1.52–3.50)	<0.001	1.58 (1.00–2.48)	0.048

^1^ Adjusted for baseline demographic and clinical characteristics, baseline laboratory measures including hematocrit, platelet count, Fibrosis-4 index, and estimated glomerular filtration rate, and time-varying concomitant medications.

## Data Availability

The data that support the findings of this study are not publicly available because they contain potentially identifiable patient-level information from four participating hospitals. Data may be available from the corresponding authors upon reasonable request, subject to institutional and ethical approvals. The analytic code used in this study is available from the corresponding authors upon reasonable request.

## Supplementary Materials

The following supporting information can be downloaded at: https://www.mdpi.com/article/10.3390/jcm15103590/s1, Table S1: Variable-level missingness and multiple imputation in the primary analysis cohort; Table S2: Variable-level missingness and multiple imputation in the cohort without prior proton pump inhibitor exposure; Table S3: Proportional hazards assumption diagnostics for the primary time-dependent Cox models; Table S4: STROBE Statement—Checklist of items that should be included in reports of cohort studies; Figure S1: Multiple-imputation diagnostics in the primary analysis cohort; Figure S2: Robustness of the association between concomitant proton pump inhibitor use and gastrointestinal bleeding outcomes during oral anticoagulant therapy; Figure S3: Covariate balance before and after inverse probability of treatment weighting in the time-varying analysis; Figure S4: Exploratory subgroup analyses of the associations between 7-day lagged time-varying proton pump inhibitor exposure and clinical outcomes; Figure S5: Event-time clustering of first observed proton pump inhibitor initiation before gastrointestinal bleeding outcomes.
