# Supplementary material for: Proton Pump Inhibitor Use and Clinical Outcomes in Atrial Fibrillation During Anticoagulation
Source: J Clin Med. 2026 May 8;15(10):3590. doi: 10.3390/jcm15103590 (PMC13207872; doi:10.3390/jcm15103590)
Supplement: Supplementary file 1 [file jcm-15-03590-s001.zip › jcm-4294169-Supplementary.pdf]

Supplementary Materials

Proton Pump Inhibitor Use and Clinical Outcomes in Atrial Fibrillation During Anticoagulation

Contents

Table S1. Variable-level missingness and multiple imputation in the primary analysis cohort

Table S2. Variable-level missingness and multiple imputation in the cohort without prior proton pump inhibitor exposure

Table S3. Proportional hazards assumption diagnostics for the primary time-dependent Cox models

Table S4. STROBE Statement—Checklist of items that should be included in reports of cohort studies

Figure S1. Multiple-imputation diagnostics in the primary analysis cohort

Figure S2. Robustness of the association between concomitant proton pump inhibitor use and gastrointestinal bleeding outcomes during oral anticoagulant therapy

Figure S3. Covariate balance before and after inverse probability of treatment weighting in the time-varying analysis

Figure S4. Exploratory subgroup analyses of the associations between 7-day lagged time-varying proton pump inhibitor exposure and clinical outcomes

Figure S5. Event-time clustering of first observed proton pump inhibitor initiation before gastrointestinal bleeding outcomes.

Table S1. Variable-level missingness and multiple imputation in the primary analysis cohort

Variables	Missing, n (%)	Role in multiple imputation	Used in final outcome model	Observed summary	Imputed summary
Proton pump inhibitor at baseline	0 (0%)	Predictor only	No	3211 (28.7%)	Not imputed
Composite outcome indicator	0 (0%)	Predictor only	No	705 (6.3%)	Not imputed
Time to composite outcome/censoring	0 (0%)	Predictor only	No	209.00 [56.00, 629.00]	Not imputed
Age, years	0 (0%)	Predictor only	Yes	71.00 [62.00, 78.00]	Not imputed
Female	0 (0%)	Predictor only	Yes	4743 (42.3%)	Not imputed
Height, cm	1398 (12.5%)	Imputed	No	163.0 [155.0, 170.0]	163.0 [155.0, 170.0]
Weight, kg	995 (8.9%)	Imputed	No	64.0 [55.8, 72.3]	65.0 [57.0, 74.0]

Heart failure	0 (0%)	Predictor only	Yes	2975 (26.6%)	Not imputed
Hypertension	0 (0%)	Predictor only	Yes	5226 (46.6%)	Not imputed
Diabetes mellitus	0 (0%)	Predictor only	Yes	2205 (19.7%)	Not imputed
Dyslipidemia	0 (0%)	Predictor only	Yes	1485 (13.3%)	Not imputed
Peripheral artery disease	0 (0%)	Predictor only	Yes	123 (1.1%)	Not imputed
Coronary artery disease / myocardial infarction	0 (0%)	Predictor only	Yes	976 (8.7%)	Not imputed
Prior thromboembolic event	0 (0%)	Predictor only	Yes	2686 (24.0%)	Not imputed
Prior bleeding event	0 (0%)	Predictor only	Yes	480 (4.3%)	Not imputed
Antiplatelet drug at baseline	0 (0%)	Predictor only	No	3540 (31.6%)	Not imputed
Diuretic drug at baseline	0 (0%)	Predictor only	No	4069 (36.3%)	Not imputed

Nonsteroidal anti-inflammatory drug at baseline	0 (0%)	Predictor only	No	1557 (13.9%)	Not imputed
Renin-angiotensin system inhibitor at baseline	0 (0%)	Predictor only	No	4284 (38.2%)	Not imputed
Beta-blocker at baseline	0 (0%)	Predictor only	No	4390 (39.2%)	Not imputed
Statin at baseline	0 (0%)	Predictor only	No	4364 (39.0%)	Not imputed
Hematocrit, %	1349 (12.0%)	Imputed	Yes	39.8 [35.0, 43.9]	41.6 [37.4, 45.3]
Platelet count, $\times 10^9$/L	1348 (12.0%)	Imputed	Yes	198.0 [160.0, 242.0]	201.0 [163.0, 245.0]
Aspartate aminotransferase, IU	968 (8.6%)	Imputed	No	26.0 [21.0, 34.0]	25.0 [20.0, 32.0]
Alanine aminotransferase, IU	1884 (16.8%)	Imputed	No	20.0 [14.0, 30.0]	20.0 [14.0, 29.0]
Creatinine, mg/dL	438 (3.9%)	Imputed	No	0.87 [0.72, 1.06]	0.86 [0.71, 1.04]

Table S2. Variable-level missingness and multiple imputation in the cohort without prior proton pump inhibitor exposure

Variables	Missing, n (%)	Role in multiple imputation	Used in final outcome model	Observed summary	Imputed summary
Proton pump inhibitor at baseline	0 (0%)	Predictor only	No	1444 (16.5%)	Not imputed
Composite outcome indicator	0 (0%)	Predictor only	No	511 (5.8%)	Not imputed
Time to composite outcome/censoring	0 (0%)	Predictor only	No	223.00 [60.00, 652.75]	Not imputed
Age, years	0 (0%)	Predictor only	Yes	70.00 [61.00, 78.00]	Not imputed
Female	0 (0%)	Predictor only	Yes	3595 (41.0%)	Not imputed
Height, cm	1323 (15.1%)	Imputed	No	163.4 [155.0, 170.0]	163.0 [155.0, 170.0]

Weight, kg	947 (10.8%)	Imputed	No	65.0 [56.1, 73.0]	65.2 [57.0, 74.0]
Heart failure	0 (0%)	Predictor only	Yes	2268 (25.9%)	Not imputed
Hypertension	0 (0%)	Predictor only	Yes	4131 (47.2%)	Not imputed
Diabetes mellitus	0 (0%)	Predictor only	Yes	1658 (18.9%)	Not imputed
Dyslipidemia	0 (0%)	Predictor only	Yes	1165 (13.3%)	Not imputed
Peripheral artery disease	0 (0%)	Predictor only	Yes	80 (0.9%)	Not imputed
Coronary artery disease / myocardial infarction	0 (0%)	Predictor only	Yes	673 (7.7%)	Not imputed
Prior thromboembolic event	0 (0%)	Predictor only	Yes	1716 (19.6%)	Not imputed
Prior bleeding event	0 (0%)	Predictor only	Yes	253 (2.9%)	Not imputed
Antiplatelet drug at baseline	0 (0%)	Predictor only	No	2544 (29.0%)	Not imputed
Diuretic drug at baseline	0 (0%)	Predictor only	No	3018 (34.5%)	Not imputed

Nonsteroidal anti-inflammatory drug at baseline	0 (0%)	Predictor only	No	996 (11.4%)	Not imputed
Renin-angiotensin system inhibitor at baseline	0 (0%)	Predictor only	No	3365 (38.4%)	Not imputed
Beta-blocker at baseline	0 (0%)	Predictor only	No	3390 (38.7%)	Not imputed
Statin at baseline	0 (0%)	Predictor only	No	3149 (36.0%)	Not imputed
Hematocrit, %	1265 (14.4%)	Imputed	Yes	40.6 [36.1, 44.4]	41.8 [37.6, 45.4]
Platelet count, $\times 10^9$/L	1264 (14.4%)	Imputed	Yes	199.0 [161.0, 241.0]	202.0 [164.0, 245.0]
Aspartate aminotransferase, IU	919 (10.5%)	Imputed	No	26.0 [21.0, 34.0]	25.0 [21.0, 32.0]
Alanine aminotransferase, IU	1681 (19.2%)	Imputed	No	20.0 [14.0, 30.0]	19.0 [14.0, 28.0]
Creatinine, mg/dL	403 (4.6%)	Imputed	No	0.89 [0.73, 1.06]	0.88 [0.73, 1.04]

Table S3. Proportional hazards assumption diagnostics for the primary time-dependent Cox models

Outcome	Global test, median p value (range)	Global p < 0.05, n/30	Main exposure test, median p value (range)	Main exposure p < 0.05, n/30	Interpretation
Composite outcome	<0.001 (<0.001-<0.001)	30/30	0.003 (0.003-0.004)	30/30	Possible non-proportionality
Thromboembolic event	<0.001 (<0.001-<0.001)	30/30	<0.001 (<0.001-<0.001)	30/30	Possible non-proportionality
Major bleeding event	0.302 (0.160-0.414)	0/30	0.363 (0.329-0.384)	0/30	No consistent violation signal
Gastrointestinal bleeding, total	0.733 (0.572-0.817)	0/30	0.880 (0.795-0.910)	0/30	No consistent violation signal
Upper gastrointestinal bleeding	0.910 (0.858-0.954)	0/30	0.709 (0.693-0.730)	0/30	No consistent violation signal
Lower gastrointestinal bleeding	0.215 (0.082-0.451)	0/30	0.092 (0.087-0.103)	0/30	No consistent violation signal
Gastrointestinal bleeding of undetermined source	0.743 (0.716-0.786)	0/30	0.537 (0.505-0.598)	0/30	No consistent violation signal
All-cause mortality	0.102 (0.035-0.172)	3/30	0.742 (0.708-0.836)	0/30	No consistent violation signal

The proportional hazards assumption was assessed using scaled Schoenfeld residuals across the 30 imputed datasets. The global test refers to the overall model-level proportional hazards test. The main exposure test refers to the test for 7-day lagged time-varying proton pump inhibitor

exposure. A possible non-proportionality signal was defined when the test for the main exposure showed $p < 0.05$ in at least half of the imputed datasets.

Table S4. STROBE Statement—Checklist of items that should be included in reports of ***cohort studies***

	Item No	Recommendation	Page No
**Title and abstract**	1	(*a*) Indicate the study's design with a commonly used term in the title or the abstract	3
		(*b*) Provide in the abstract an informative and balanced summary of what was done and what was found	3-4
**Introduction**			
Background/rationale	2	Explain the scientific background and rationale for the investigation being reported	5
Objectives	3	State specific objectives, including any prespecified hypotheses	5
**Methods**			
Study design	4	Present key elements of study design early in the paper	6-10
Setting	5	Describe the setting, locations, and relevant dates, including periods of recruitment, exposure, follow-up, and data collection	6-7
Participants	6	(*a*) Give the eligibility criteria, and the sources and methods of selection of participants. Describe methods of follow-up	6-7
		(*b*) For matched studies, give matching criteria and number of exposed and unexposed	NA
Variables	7	Clearly define all outcomes, exposures, predictors, potential confounders, and effect modifiers. Give diagnostic criteria, if applicable	7-9
Data sources/ measurement	8*	For each variable of interest, give sources of data and details of methods of assessment (measurement). Describe comparability of assessment methods if there is more than one group	6-9
Bias	9	Describe any efforts to address potential sources of bias	7-10

Study size	10	Explain how the study size was arrived at	6-7
Quantitative variables	11	Explain how quantitative variables were handled in the analyses. If applicable, describe which groupings were chosen and why	9-10
Statistical methods	12	(*a*) Describe all statistical methods, including those used to control for confounding	10
		(*b*) Describe any methods used to examine subgroups and interactions	10
		(*c*) Explain how missing data were addressed	9; Suppl. Table S1-S2, Fig. S1
		(*d*) If applicable, explain how loss to follow-up was addressed	7, 10
		(*e*) Describe any sensitivity analyses	10, 12-13
**Results**			
Participants	13*	(a) Report numbers of individuals at each stage of study—eg numbers potentially eligible, examined for eligibility, confirmed eligible, included in the study, completing follow-up, and analysed	11; Fig. 1
		(b) Give reasons for non-participation at each stage	Fig. 1
		(c) Consider use of a flow diagram	Fig. 1
Descriptive data	14*	(a) Give characteristics of study participants (eg demographic, clinical, social) and information on exposures and potential confounders	11; Table 1
		(b) Indicate number of participants with missing data for each variable of interest	11; Suppl. Table S1-S2
		(c) Summarise follow-up time (eg, average and total amount)	30-31; Table 2
Outcome data	15*	Report numbers of outcome events or summary measures over time	11; 30-31; Table 2

Main results	16	(*a*) Give unadjusted estimates and, if applicable, confounder-adjusted estimates and their precision (eg, 95% confidence interval). Make clear which confounders were adjusted for and why they were included	30-31; Table 2

		(*b*) Report category boundaries when continuous variables were categorized	NA
		(*c*) If relevant, consider translating estimates of relative risk into absolute risk for a meaningful time period	NA
Other analyses	17	Report other analyses done—eg analyses of subgroups and interactions, and sensitivity analyses	10-13; Fig. 2; Suppl. Fig. S2-S5
**Discussion**			
Key results	18	Summarise key results with reference to study objectives	13-16
Limitations	19	Discuss limitations of the study, taking into account sources of potential bias or imprecision. Discuss both direction and magnitude of any potential bias	15-16
Interpretation	20	Give a cautious overall interpretation of results considering objectives, limitations, multiplicity of analyses, results from similar studies, and other relevant evidence	13-16
Generalisability	21	Discuss the generalisability (external validity) of the study results	15-16
**Other information**			
Funding	22	Give the source of funding and the role of the funders for the present study and, if applicable, for the original study on which the present article is based	17

*Give information separately for exposed and unexposed groups.

Note: An Explanation and Elaboration article discusses each checklist item and gives methodological background and published examples of transparent reporting. The STROBE checklist is best used in conjunction with this article (freely available on the Web sites of PLoS Medicine at http://www.plosmedicine.org/, Annals of Internal Medicine at http://www.annals.org/, and Epidemiology at http://www.epidem.com/). Information on the STROBE Initiative is available at http://www.strobe-statement.org.

Figure S1. Multiple-imputation diagnostics in the primary analysis cohort

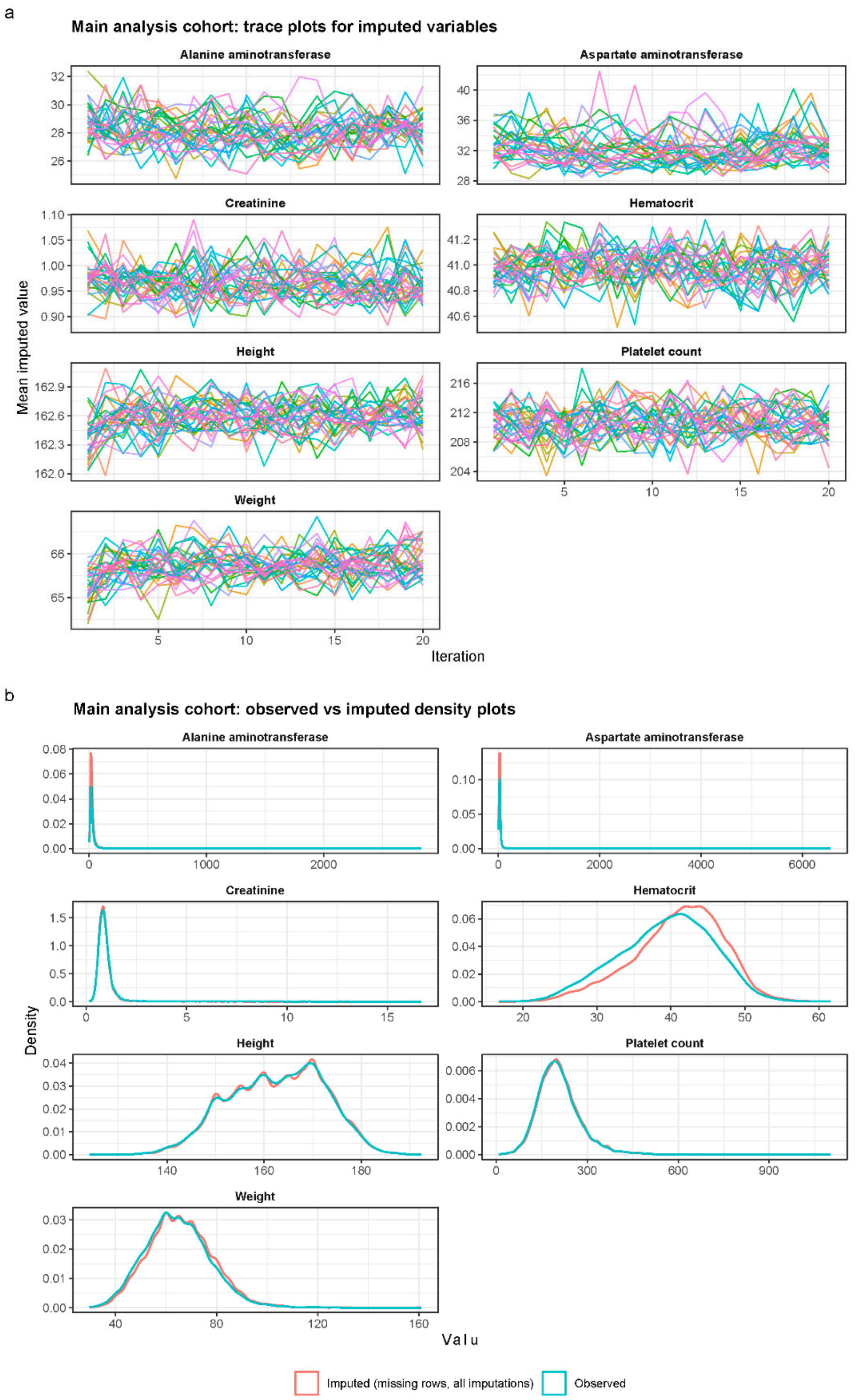

Trace plots for imputed variables in the primary analysis cohort (a) and density plots comparing observed values with imputed values among observations that were originally missing in the primary analysis cohort (b). Overall, the trace plots showed no marked drift over iterations, and the observed and imputed distributions were broadly similar, supporting the overall stability and plausibility of the imputation procedure.

Figure S2. Robustness of the association between concomitant proton pump inhibitor use and gastrointestinal bleeding outcomes during oral anticoagulant therapy

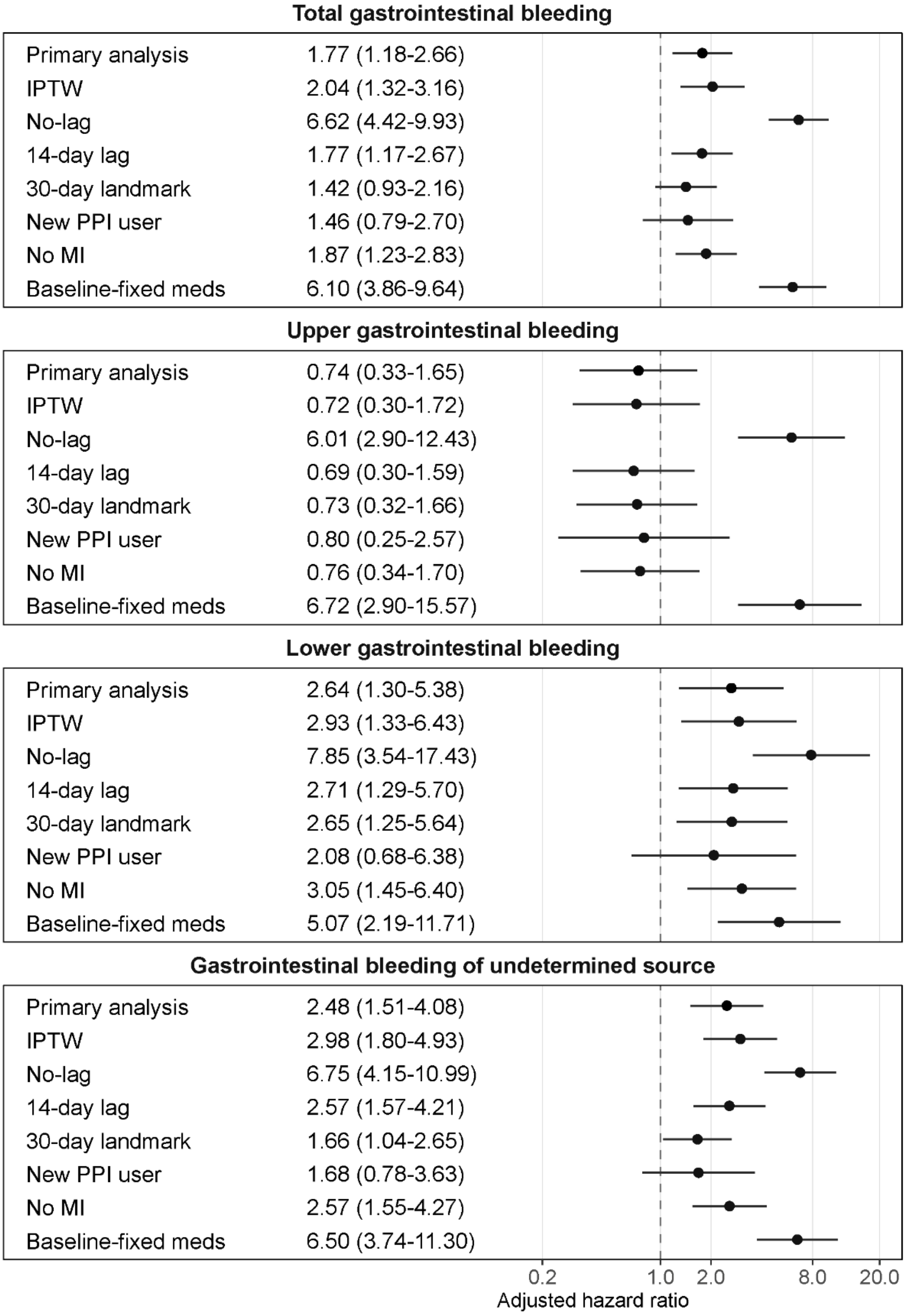

Adjusted hazard ratios and 95% confidence intervals for the association between concomitant proton pump inhibitor use and four gastrointestinal bleeding outcomes during oral anticoagulant therapy. The figure compares the primary analysis with robustness analyses using alternative analytic strategies. Consistency across analyses supports robustness, whereas variation across analyses suggests greater sensitivity of the estimate. All estimates should be interpreted as observational associations.

Figure S3. Covariate balance before and after inverse probability of treatment weighting in the time-varying analysis

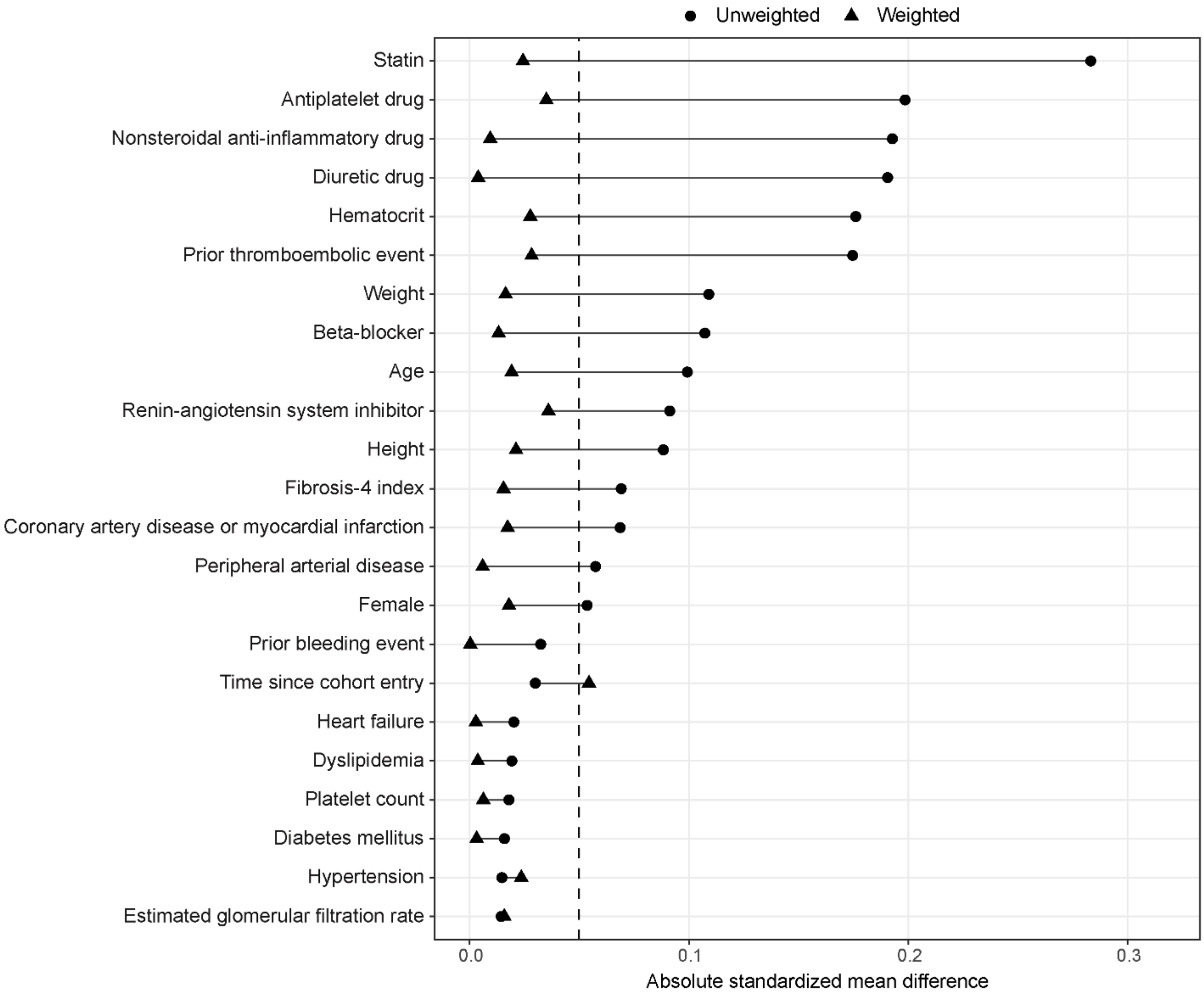

Love plot showing absolute standardized mean differences for all covariates before and after time-varying inverse probability of treatment weighting for the selected propensity score specification. For each covariate, absolute standardized mean differences were calculated within each imputed dataset and summarized across imputations using the median. Points represent unweighted and weighted values, and the dashed vertical line indicates the prespecified balance threshold (absolute standardized mean difference = 0.05).

Figure S4. Exploratory subgroup analyses of the associations between 7-day lagged time-varying proton pump inhibitor exposure and clinical outcomes

a

Composite outcome

Subgroup	HR (95% CI)	P for interaction
Age		0.036
<65 years	1.70 (1.11–2.58)	
65–<75 years	0.94 (0.65–1.36)	
≥75 years	1.48 (1.16–1.90)	
Sex		0.839
Female	1.43 (1.10–1.85)	
Male	1.28 (0.99–1.65)	
Heart failure		0.135
No	1.47 (1.18–1.84)	
Yes	1.12 (0.82–1.54)	
Hypertension		0.219
No	1.25 (0.97–1.60)	
Yes	1.48 (1.13–1.94)	
Diabetes		0.558
No	1.33 (1.09–1.64)	
Yes	1.37 (0.92–2.02)	
Dyslipidemia		0.524
No	1.34 (1.10–1.63)	
Yes	1.44 (0.89–2.33)	
Coronary artery disease		0.767
No	1.36 (1.12–1.65)	
Yes	1.31 (0.80–2.14)	
Prior thromboembolic event		0.609
No	1.33 (1.04–1.69)	
Yes	1.33 (1.01–1.75)	
Prior major bleeding event		0.917
No	1.36 (1.13–1.64)	
Yes	1.14 (0.49–2.69)	
Hematocrit at baseline		0.487
≥22%	1.37 (1.05–1.78)	
<22%	1.30 (1.01–1.68)	
Fibrosis-4 index at baseline		0.414
Low-intermediate	1.32 (0.99–1.76)	
High	1.40 (1.11–1.77)	
Estimated GFR at baseline		0.462
≥60 mL/min/1.73 m²	1.36 (1.10–1.70)	
45–<60 mL/min/1.73 m²	1.57 (0.99–2.49)	
30–<45 mL/min/1.73 m²	1.23 (0.58–2.61)	
<30 mL/min/1.73 m²	0.88 (0.43–1.79)	
OAC class at baseline		0.737
Warfarin	1.65 (1.25–2.19)	
NOAC	1.42 (1.09–1.84)	
Antiplatelet at baseline		0.940
No	1.32 (1.02–1.71)	
Yes	1.47 (1.14–1.90)	
Diuretic at baseline		0.986
No	1.31 (1.00–1.71)	
Yes	1.40 (1.10–1.78)	
NSAID at baseline		0.651
No	1.27 (1.02–1.59)	
Yes	1.52 (1.11–2.09)	
RAS inhibitor at baseline		0.253
No	1.26 (0.98–1.63)	
Yes	1.50 (1.16–1.95)	
Beta-blocker at baseline		0.514
No	1.45 (1.13–1.85)	
Yes	1.27 (0.98–1.65)	
Statin at baseline		0.779
No	1.25 (0.93–1.69)	
Yes	1.42 (1.13–1.78)	

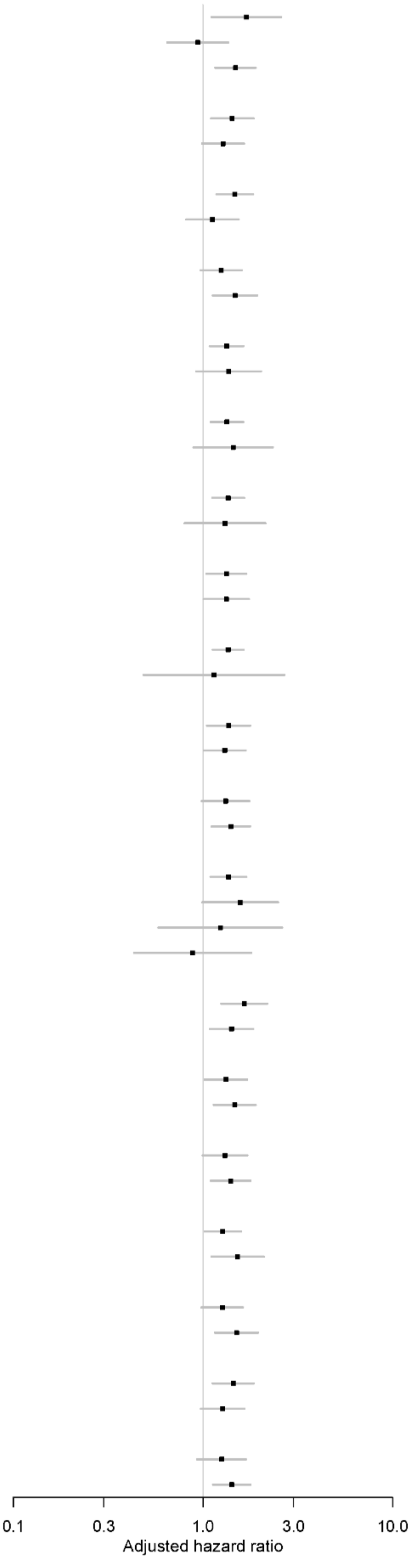

b

Thromboembolism

Subgroup	HR (95% CI)	P for interaction
Age		0.329
<65 years	1.28 (0.71–2.29)	
65–<75 years	0.82 (0.50–1.35)	
≥75 years	1.10 (0.76–1.61)	
Sex		0.111
Female	1.31 (0.92–1.87)	
Male	0.81 (0.54–1.20)	
Heart failure		0.837
No	1.02 (0.74–1.41)	
Yes	1.14 (0.72–1.78)	
Hypertension		0.436
No	0.96 (0.68–1.37)	
Yes	1.19 (0.79–1.79)	
Diabetes		0.841
No	1.05 (0.79–1.41)	
Yes	1.06 (0.56–2.01)	
Dyslipidemia		0.779
No	1.05 (0.79–1.39)	
Yes	1.07 (0.51–2.24)	
Coronary artery disease		0.931
No	1.04 (0.78–1.38)	
Yes	1.23 (0.62–2.44)	
Prior thromboembolic event		0.013
No	1.28 (0.92–1.79)	
Yes	0.80 (0.53–1.20)	
Prior major bleeding event		0.327
No	1.09 (0.84–1.43)	
Yes	0.25 (0.05–1.36)	
Hematocrit at baseline		0.924
≥22%	1.04 (0.72–1.49)	
<22%	1.08 (0.73–1.59)	
Fibrosis-4 index at baseline		0.933
Low-intermediate	0.92 (0.60–1.41)	
High	1.18 (0.84–1.65)	
Estimated GFR at baseline		0.244
≥60 mL/min/1.73 m²	0.90 (0.65–1.25)	
45–<60 mL/min/1.73 m²	1.86 (1.02–3.40)	
30–<45 mL/min/1.73 m²	1.14 (0.37–3.49)	
<30 mL/min/1.73 m²	0.70 (0.19–2.53)	
OAC class at baseline		0.321
Warfarin	1.52 (1.04–2.22)	
NOAC	0.97 (0.65–1.45)	
Antiplatelet at baseline		0.941
No	0.97 (0.66–1.41)	
Yes	1.26 (0.87–1.83)	
Diuretic at baseline		0.278
No	0.88 (0.60–1.27)	
Yes	1.26 (0.87–1.83)	
NSAID at baseline		0.720
No	0.97 (0.71–1.34)	
Yes	1.11 (0.71–1.72)	
RAS inhibitor at baseline		0.128
No	0.92 (0.63–1.32)	
Yes	1.31 (0.90–1.91)	
Beta-blocker at baseline		0.690
No	1.09 (0.75–1.57)	
Yes	0.99 (0.68–1.45)	
Statin at baseline		0.968
No	1.00 (0.64–1.56)	
Yes	1.10 (0.79–1.52)	

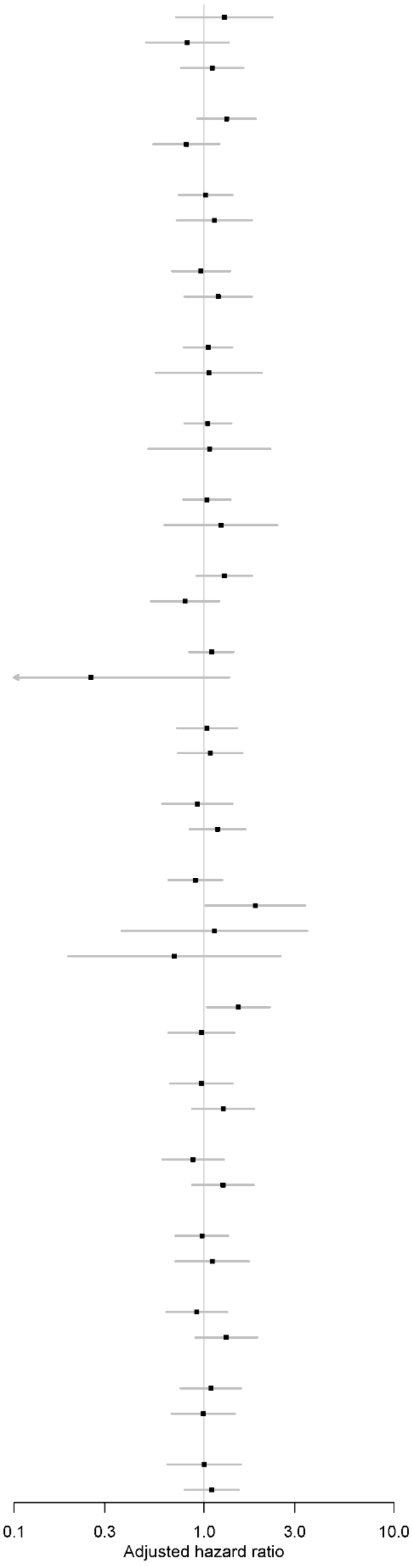

c

Major bleeding

Subgroup	HR (95% CI)	P for interaction
Age		0.043
<65 years	2.65 (1.36–5.15)	
65–<75 years	1.05 (0.57–1.92)	
≥75 years	1.98 (1.37–2.86)	
Sex		0.835
Female	1.84 (1.24–2.73)	
Male	1.81 (1.22–2.68)	
Heart failure		0.071
No	2.21 (1.58–3.09)	
Yes	1.19 (0.73–1.95)	
Hypertension		0.973
No	1.93 (1.33–2.79)	
Yes	1.75 (1.14–2.69)	
Diabetes		0.743
No	1.92 (1.39–2.64)	
Yes	1.38 (0.74–2.57)	
Dyslipidemia		0.874
No	1.90 (1.41–2.55)	
Yes	1.56 (0.71–3.42)	
Coronary artery disease		0.476
No	1.91 (1.42–2.57)	
Yes	1.46 (0.69–3.07)	
Prior thromboembolic event		0.012
No	1.38 (0.92–2.06)	
Yes	2.60 (1.70–3.98)	
Prior major bleeding event		0.419
No	1.79 (1.34–2.39)	
Yes	5.56 (1.13–27.28)	
Hematocrit at baseline		0.507
≥22%	1.80 (1.18–2.72)	
<22%	1.75 (1.18–2.59)	
Fibrosis-4 index at baseline		0.055
Low-intermediate	2.21 (1.44–3.38)	
High	1.64 (1.13–2.39)	
Estimated GFR at baseline		0.122
≥60 mL/min/1.73 m²	2.33 (1.68–3.23)	
45–<60 mL/min/1.73 m²	1.15 (0.53–2.50)	
30–<45 mL/min/1.73 m²	1.47 (0.52–4.22)	
<30 mL/min/1.73 m²	0.83 (0.33–2.12)	
OAC class at baseline		0.318
Warfarin	2.08 (1.32–3.28)	
NOAC	2.20 (1.49–3.26)	
Antiplatelet at baseline		0.476
No	1.79 (1.19–2.69)	
Yes	1.91 (1.30–2.79)	
Diuretic at baseline		0.070
No	2.43 (1.57–3.75)	
Yes	1.51 (1.04–2.18)	
NSAID at baseline		0.101
No	1.60 (1.13–2.27)	
Yes	2.71 (1.62–4.55)	
RAS inhibitor at baseline		0.900
No	1.76 (1.19–2.62)	
Yes	1.89 (1.28–2.79)	
Beta-blocker at baseline		0.796
No	1.92 (1.30–2.84)	
Yes	1.83 (1.25–2.69)	
Statin at baseline		0.680
No	1.93 (1.23–3.03)	
Yes	1.73 (1.22–2.45)	

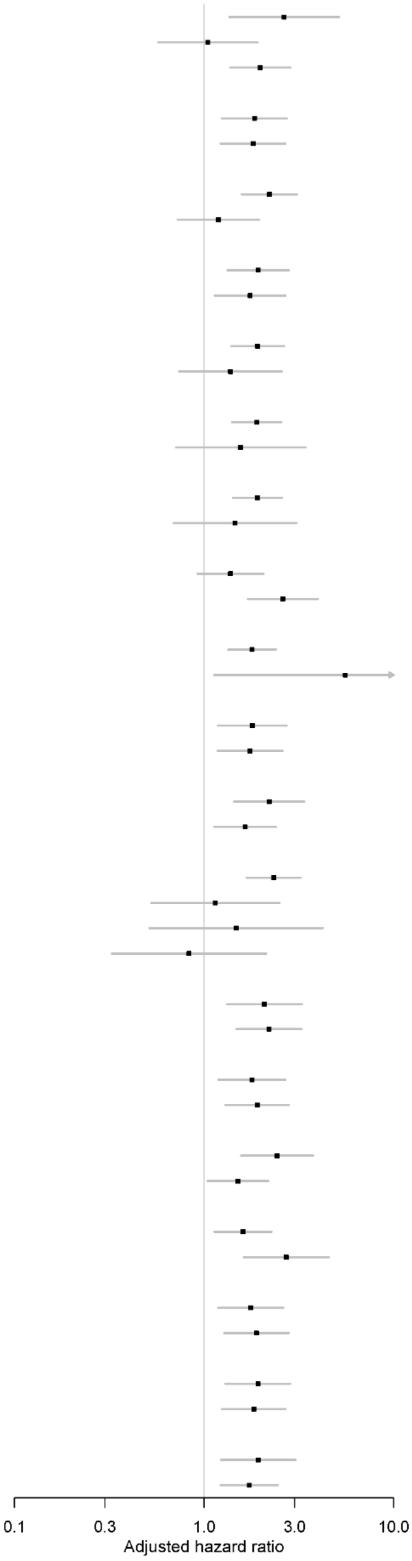

d

All-cause mortality

Subgroup	HR (95% CI)	P for interaction
Age		0.442
<65 years	4.89 (1.55–15.43)	
65–<75 years	1.27 (0.48–3.40)	
≥75 years	1.43 (0.82–2.49)	
Sex		0.606
Female	1.39 (0.69–2.78)	
Male	1.67 (0.91–3.07)	
Heart failure		0.375
No	1.81 (0.97–3.35)	
Yes	1.28 (0.66–2.50)	
Hypertension		0.299
No	1.16 (0.55–2.45)	
Yes	1.92 (1.08–3.43)	
Diabetes		0.246
No	1.29 (0.74–2.26)	
Yes	2.48 (1.11–5.55)	
Dyslipidemia		0.120
No	1.28 (0.75–2.18)	
Yes	3.45 (1.39–8.52)	
Coronary artery disease		0.250
No	1.37 (0.81–2.33)	
Yes	2.35 (0.88–6.30)	
Prior thromboembolic event		0.413
No	1.73 (0.99–3.01)	
Yes	1.26 (0.59–2.69)	
Prior major bleeding event		0.794
No	1.55 (0.96–2.50)	
Yes	1.82 (0.23–14.37)	
Hematocrit at baseline		0.189
≥22%	1.92 (0.94–3.94)	
<22%	1.27 (0.71–2.26)	
Fibrosis-4 index at baseline		0.433
Low-intermediate	2.06 (0.91–4.63)	
High	1.41 (0.80–2.46)	
Estimated GFR at baseline		0.443
≥60 mL/min/1.73 m²	1.63 (0.92–2.90)	
45–<60 mL/min/1.73 m²	0.66 (0.12–3.67)	
30–<45 mL/min/1.73 m²	2.18 (0.38–12.45)	
<30 mL/min/1.73 m²	2.11 (0.61–7.38)	
OAC class at baseline		0.876
Warfarin	1.89 (0.92–3.87)	
NOAC	1.45 (0.75–2.82)	
Antiplatelet at baseline		0.233
No	1.92 (1.06–3.49)	
Yes	1.19 (0.60–2.36)	
Diuretic at baseline		0.899
No	1.56 (0.56–4.40)	
Yes	1.51 (0.92–2.49)	
NSAID at baseline		0.260
No	1.87 (1.12–3.12)	
Yes	1.04 (0.42–2.54)	
RAS inhibitor at baseline		0.592
No	1.43 (0.75–2.72)	
Yes	1.77 (0.92–3.40)	
Beta-blocker at baseline		0.509
No	1.95 (1.08–3.51)	
Yes	1.19 (0.59–2.40)	
Statin at baseline		0.389
No	1.16 (0.57–2.34)	
Yes	1.87 (1.01–3.46)	

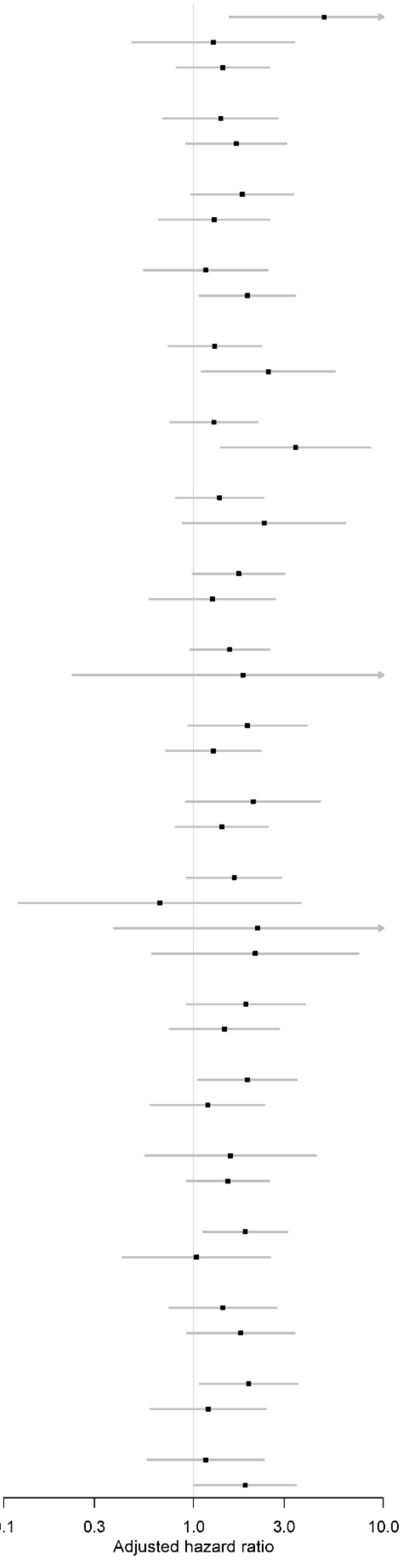

Forest plots for the clinical outcomes are shown as follows: composite outcome (a), thromboembolism (b), major bleeding (c), and all-cause mortality (d). Squares indicate adjusted hazard ratios and horizontal lines indicate 95% confidence intervals. P for interaction values are shown for each subgroup.

Abbreviations: GFR, glomerular filtration rate; OAC, oral anticoagulant; NSAID, nonsteroidal anti-inflammatory drug; RAS, renin-angiotensin system.

Figure S5. Event-time clustering of first observed proton pump inhibitor initiation before gastrointestinal bleeding outcomes.

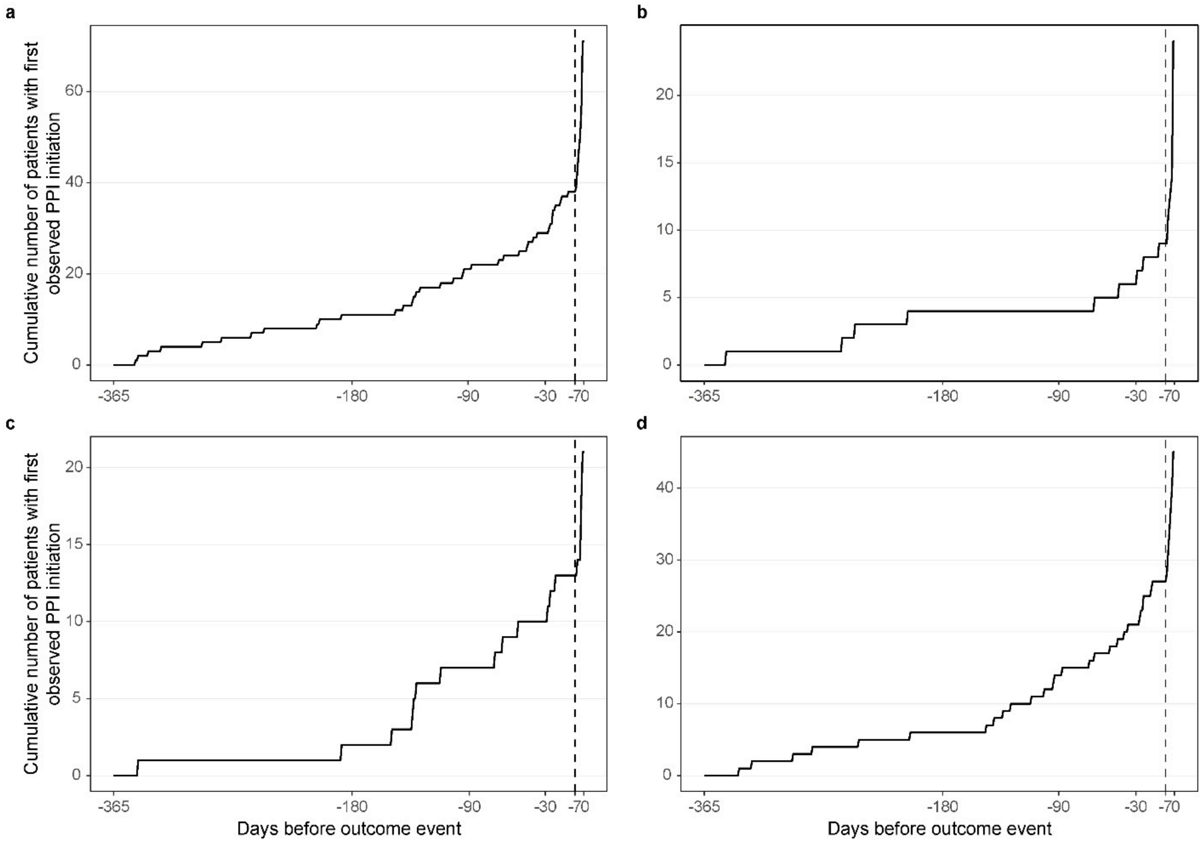

Panels show the cumulative number of patients with a first observed proton pump inhibitor initiation within 365 days before the outcome event for overall gastrointestinal bleeding (a), upper gastrointestinal bleeding (b), lower gastrointestinal bleeding (c), and gastrointestinal bleeding of undetermined source (d). The dashed vertical line indicates day −7, which corresponds to the primary lag used in the primary analysis.
